# Drain Fluid Amylase as an Early Negative Predictor of Salivary Fistula Following Free Flap Reconstruction

**DOI:** 10.1002/micr.70066

**Published:** 2025-05-07

**Authors:** Micah K. Harris, Mark Kubik, Mario G. Solari, Kevin J. Contrera, Ore Odeniyi, Zoey Morton, Lauren Gardiner, Matthew E. Spector, Shaum S. Sridharan

**Affiliations:** ^1^ Department of Otolaryngology—Head and Neck Surgery University of Pittsburgh Medical Center Pittsburgh Pennsylvania USA; ^2^ Department of Plastic Surgery University of Pittsburgh Medical Center Pittsburgh Pennsylvania USA

**Keywords:** amylase, fistula, free flap, head and neck reconstruction, salivary fistula

## Abstract

**Objectives:**

Salivary fistula is a known complication following head and neck free flap reconstruction involving the aerodigestive tract. We sought to examine the association between surgical drain fluid amylase and salivary fistula formation during postoperative hospitalization.

**Methods:**

Eighty patients who underwent head and neck reconstruction involving the aerodigestive tract at our institution between 2019 and 2023 were included. Amylase concentration (IU/L) was measured from a Jackson‐Pratt drain located along the mucosal closure line on postoperative days 1–5.

**Results:**

Twelve patients (15%) developed salivary fistulas. The change in drain amylase concentration between postoperative day 1 and day 2 was found to be significantly higher in those who developed a fistula during postoperative hospitalization. A receiver operating characteristic curve found that a threshold of 15% provided a sensitivity of 58.3% and specificity of 80.6% (area under the curve 0.767) to predict salivary fistula. This threshold remained significant on multivariate analysis (odds ratio 5.35, 95% confidence interval 1.79–24.3) when controlling for prior radiation, perioperative transfusion, and total laryngectomy. When retrospectively applied to our cohort, a cutoff of 15% resulted in a positive predictive value of 35% and a negative predictive value of 91.5%.

**Conclusion:**

Change in surgical drain fluid amylase from postoperative day 1 to 2 was associated with fistula formation following free flap reconstruction of the aerodigestive tract. Importantly, a change in amylase of < 15% from postoperative day 1 to 2 was best at identifying patients who are at low risk of developing salivary fistula during postoperative hospitalization, with a negative predictive value of 91.5%.

## 1 Introduction

The development of salivary fistula is a known complication following head and neck free flap reconstruction, with the reported incidence reaching over 20% (Sayles and Grant 2014). Salivary fistulas may form after any surgical ablation that violates the mucosal salivary barrier, including total laryngectomy, or resection of large oral cavity, oropharyngeal, and hypopharyngeal tumors. The introduction of saliva into the previously sterile neck results in tissue inflammation, infection, and necrosis. Salivary fistulas are typically suspected based on clinical evaluation, with signs including new swelling, erythema, tenderness, leukocytosis, fever, increased drain output, or frank saliva in the drain fluid. A suspected fistula may be treated with antibiotics and bedside packing, or if conservative management fails, then surgical repair may be pursued (Kiong et al. 2017; White et al. 2012). Salivary fistulas often develop while still in the postoperative hospitalization period, leading to prolonged length of stay, increased hospital costs, and concurrently increased risk of developing further complications. Hence, accurately predicting formation of salivary fistulas is of high importance. Conversely, the ability to identify patients who are at low risk of developing salivary fistula during hospitalization is of equal value, as this can allow for accelerated discharge and rehabilitation while maintaining patient safety.

Many prior studies have correlated salivary fistula formation with different variables such as preoperative radiation therapy (RT), malnutrition, transfusion requirement, and hypothyroidism (Lansaat et al. 2018; Tassone et al. 2022; White et al. 2012). However, these markers often lack quantifiability and clinical applicability. Amylase is a digestive enzyme that is predominantly secreted by the pancreas and salivary glands. Surgical drain amylase has been studied extensively in the field of pancreatic surgery and is commonly used in the postoperative setting to rule out pancreatic fistula following pancreatic resection (Israel et al. 2014; Liu et al. 2018; Yang et al. 2015). However, few studies have investigated the association of surgical drain amylase and salivary fistula formation following head and neck reconstruction (Aydoğan et al. 2003; Kasapoğlu et al. 2009; Larsen and Schuller 1984; Morton et al. 2007). Moreover, only one study within head and neck has reported a quantifiable amylase value predictive of salivary fistula, leading to confusion regarding the clinical relevance of amylase. Here, we assessed the association between surgical drain amylase and salivary fistula development in patients who underwent head and neck free flap reconstruction for cancer, highlighting the ability of amylase to identify patients at low risk of developing fistula during postoperative hospitalization.

## 2 Methods

We collected data on 80 patients who underwent free flap reconstruction for head and neck cancer defects between August 2019 and October 2023 at the University of Pittsburgh Medical Center (UPMC). Approval was obtained through the UPMC Institutional Review Board. Data were retrospectively collected. A third party collected amylase values, and the attending surgeons were not made aware of what the amylase values were. In this way, we deliberately did not modify clinical management based on amylase levels, in order to better understand how amylase values might predict outcomes.

Surgical drain fluid amylase concentration (IU/L) was collected for up to five consecutive postoperative days (PODs). Each surgeon followed the same protocol of placing a Jackson‐Pratt (JP) neck drain along the mucosal closure line at the end of surgery. All subsequent amylase labs were drawn from this same JP drain. Daily (24 h) drain fluid volume was also measured from this same drain. Each defect was repaired by free tissue transfer. All included patients had at least two consecutive PODs of drain fluid amylase collected. All patients had a surgical defect that violated the salivary mucosal barrier. Drain amylase concentrations were determined by removing all the fluid in the JP drain system at approximately 6 A.M. each day. Amylase concentration had a minimum recordable limit of 1 IU/L and maximum of 20,000 IU/L. Salivary fistula was defined by clinical exam during postoperative hospitalization, and only clinically evident salivary fistulas were included. Clinically evident salivary fistulas were defined as visible fistulas that necessitated bedside wound packing +/− operative management. Percent change in amylase concentration between consecutive PODs was calculated as $\left(\left({\mathrm{POD}}_{B}-{\mathrm{POD}}_{A}\right)/{\mathrm{POD}}_{B}\right)\times 100\%$.

Data collected included drain fluid amylase concentration, daily (24 h) drain fluid volume (mL), length of stay (LOS) surgical site, tumor category, nodal category, gender, cardiac comorbidities, diabetes, pulmonary comorbidities, body mass index (BMI), smoking history, alcohol abuse history, perioperative transfusion rate, prior history of RT, chemotherapy, or surgery, as well as preoperative hemoglobin, prealbumin, and thyroid stimulating hormone (TSH). Transfusion was performed when hemoglobin was < 7 g/dL or < 8 g/dL in those with cardiac comorbidities.

A power analysis was designed to estimate sample size. Prior studies from our surgeons demonstrated a salivary fistula rate ranging from 15%–30% (Heft Neal et al. 2024; Hoesli et al. 2019). Given that our cohort included oral cavity tumors which generally carry a lower risk of fistula formation than total laryngectomies, we used a predicted salivary fistula incidence of 15%. Given a confidence interval of 95% and a confidence interval width of 0.30, a sample size of 84 would allow us to detect an ROC AUC as low as 0.78 versus the null value of 0.50. This was a similar sample size as has previously been collected to investigate salivary fistula after head and neck free flap reconstruction (Aydoğan et al. 2003; Morton et al. 2007). Receiver operating characteristic (ROC) curves were designed to determine sensitivity, specificity, and area under the ROC curve (AUC), with salivary fistula as the binary state variable. Youden indices were used to select the optimum thresholds. Positive predictive value (PPV) and negative predictive value (NPV) were calculated for each statistically significant predictor by retrospectively applying the predictive thresholds to our cohort. Categorical variables were compared with chi‐squared tests and continuous variables with unpaired *t* tests. One‐way analysis of variance (ANOVA) compared > 2 categorical variables. Univariate logistic regressions were used to examine predictors of salivary fistula as a binary variable. Variables with a *p*‐value of < 0.2 on univariate analysis were included in multivariate logistic regression. Statistical analyses were performed with SPSS Statistics version 28.0 (IBM). Graphics were created using Prism 10 software (GraphPad, La Jolla, CA, USA). Statistical significance was determined by a *p*‐value of < 0.05.

## 3 Results

### 3.1 Demographics

Demographics are outlined in Table 1. A total of 80 patients were included (70.4% male, 63.2 ± 11.9 years), of which 12 (15%) developed salivary fistulas. Average BMI was 25.6 ± 5.8 kg/m^2^, with six patients (7.5%) meeting the criteria for underweight (BMI < 18.5) and 40 (50%) meeting criteria for overweight to obese (BMI > 25). Fifty‐two patients (65%) had a history of tobacco use, and 27 (33.8%) had a history of alcohol abuse. Nineteen patients (23.8%) received preoperative RT, and 19 (23.8%) received preoperative chemotherapy. Six patients (7.5%) had undergone prior surgery for their cancer. Fifteen patients (18.8%) underwent laryngectomy, and 65 (81.2%) underwent oral cavity cancer resection. Of those that underwent laryngectomy, all 15 were primarily laryngeal tumors, with additional involvement of the hypopharynx (6) and/or base of tongue (3). All 65 oral cavity tumor resections resulted in intraoperative communication between the neck and oral cavity. Primary tumor sites included the floor of mouth (20, 30.8%), buccal mucosa (14, 21.5%), alveolar ridge (13, 20%), and tongue (18, 27.7%). Seven of the 15 laryngectomy patients received preoperative RT (46.7%) versus 12 of the 65 oral cavity free flaps (18.9%, *p* = 022). Flap types were anterolateral thigh (43, 53.8%), fibula (22, 27.5%), osteocutaneous radial forearm (10, 12.5%), and scapula (5, 6.2%). The average length of stay (LOS) of those who developed a salivary fistula was 26.4 ± 14.5 days compared to 11.7 ± 4.9 days for those without a salivary fistula (*p* < 0.001). Overall, average LOS was 13.9 ± 8.8 days for the entire cohort.

**TABLE 1:** Demographical and perioperative variables.

Variable	Fistula (*n* = 12)	No fistula (*n* = 68)	*p*‐value
Gender (male)	9 (75%)	40 (69.1%)	0.74
Laryngectomy	6 (50%)	9 (13.2%)	**0.003**
Tumor category	3.6 ± 0.67	3.3 ± 0.87	0.19
Nodal category	0.72 ± 1.3	1.1 ± 1.3	0.33
Prior radiation	5 (41.7%)	14 (20.6%)	0.12
Prior chemotherapy	3 (25%)	12 (17.7%)	0.53
Prior surgery	0 (0%)	6 (8.8%)	0.29
Cardiac comorbidity	10 (83.3%)	46 (67.6%)	0.31
Diabetes	3 (25%)	8 (11.8%)	0.23
Vascular comorbidity	1 (8.3%)	6 (8.8%)	0.94
Pulmonary comorbidity	3 (25%)	15 (22.1%)	0.84
Body mass index (kg/m^2^)	26 ± 7.5	25.8 ± 5.5	0.9
Preoperative hemoglobin	12.4 ± 1.7	12.8 ± 1.9	0.45
Preoperative prealbumin	19.7 ± 7.3	19.1 ± 9.2	0.85
Preoperative TSH	1.9 ± 1.5	2.9 ± 5.9	0.8
Smoking history	9 (75%)	43 (62.2%)	0.47
Alcohol abuse history	6 (50%)	21 (30.9%)	0.21
Perioperative transfusion	3 (25%)	6 (8.8%)	0.12
Length of stay (days)	26.4 ± 14.5	11.7 ± 4.9	**< 0.001**

Abbreviation: TSH, thyroid stimulating hormone.

All patients had at least two consecutive PODs of drain amylase collected. Most patients (76.3%) had five consecutive PODs of amylase collected, and 92.5% had four consecutive PODs collected. Twelve patients (15%) developed salivary fistulas. Salivary fistula was more common following laryngectomy (6/15, 40%) versus oral cavity resection (*n* = 6/60, 10%, *p* = 0.003). There was no significant difference by gender, age, tumor category, nodal category, cardiac comorbidities, history of alcohol abuse, smoking history, diabetes, pulmonary comorbidities, preoperative hemoglobin, preoperative prealbumin, preoperative TSH, or number of perioperative transfusions (*p* > 0.05) (Table 1).

### 3.2 Drain Volume

There was no difference in the daily JP drain volume between those who developed a fistula versus those who did not on POD1 (69.4 ± 32.2 mL vs. 63.2 ± 40.8 mL, *p* = 0.68), POD2 (83.9 ± 32.1 mL vs. 83.5 ± 65.2 mL, *p* = 0.91), POD3 (56.7 ± 29 mL vs. 48 ± 49.2 mL, *p* = 0.63), POD4 (54.4 ± 28.9 mL vs. 36.1 ± 26 mL, *p* = 0.10), or POD5 (16.8 ± 34.9 mL vs. 17.1 ± 28.8 mL, *p* = 0.42). Moreover, there was no difference in the change in drain volume from POD1 to POD2 (+34.9 ± 58% vs. +58.4 ± 108%, *p* = 0.54), POD2 to POD3 (−21.9% ± 59.7% vs. −24.6% ± 62%, *p* = 0.89), POD3 to POD4 (+2.3 ± 60.5% vs. −23.9% ± 66%, *p* = 0.31) or POD4 to POD5 (−34.1% ± 34.2% vs. −32.8% ± 48.2%, *p* = 0.94).

### 3.3 Daily Amylase Values

**FIGURE 1:**
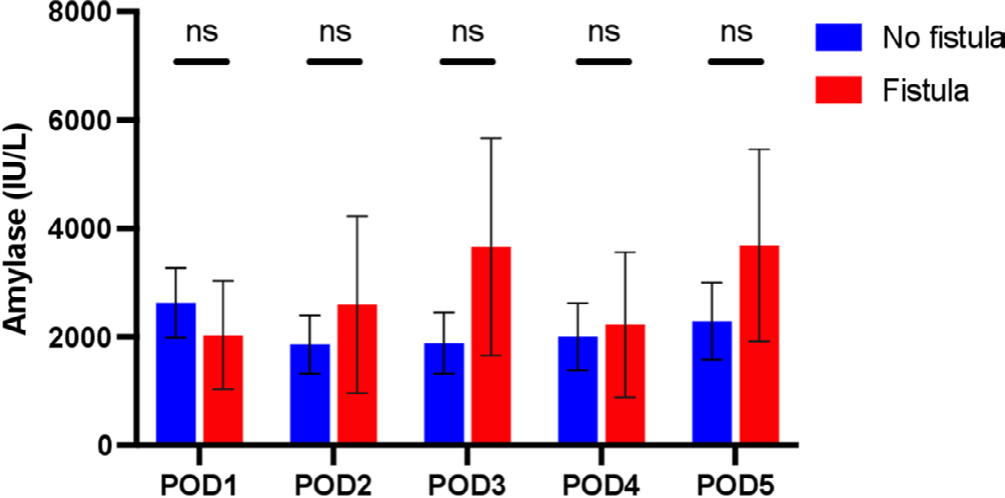
Daily amylase levels on postoperative days 1–5. ns, not significant; POD, postoperative day.

When daily drain amylase concentrations were compared between patients with a fistula versus those without a fistula, there was no difference found on any of the five PODs. On average, amylase concentrations on POD1 (fistula: 2034 ± 3460.1 vs. no fistula: 2628.5 ± 5304.6 IU/L, *p* = 0.71), POD2 (2592.2 ± 5647.2 vs. 1861,4 ± 4427.2 IU/L, *p* = 0.61), POD3 (3659.3 ± 6935.5 vs. 1886.2 ± 4614.8, *p* = 0.26), POD4 (2225.9 ± 4639.3 vs. 2003.1 ± 5108.6 IU/L, *p* = 0.89), and POD5 (3693.8 ± 6138.1 vs. 2291.7 ± 5853 IU/L, *p* = 0.49) were found to be statistically similar (Figure 1).

### 3.4 Trends in Amylase

Next, we sought to characterize trends in drain fluid amylase, as this has been suggested to be more predictive than daily values (Larsen and Schuller 1984). Percent change between two consecutive PODs was compared between those with a fistula versus those without. Percent change in drain amylase between POD2 and POD3 (∆POD3‐2, +92.8 ± 265.8% vs. +466.9% ±2911.6%, *p* = 0.66) as well as POD3 and POD4 (∆POD4‐3, +22 ± 117.1% vs. −0.9% ± 197.5%, *p* = 0.7) were statistically similar. Changes in drain amylase from POD1 to POD2 (∆POD2‐1, +98.8 ± 170% vs. −13.9% ± 70.6%, *p* < 0.001) as well as POD4 to POD5 (∆POD5‐4, +1224 ± 3650.8% vs. −29.5% ± 80.5%, *p* = 0.007) were significantly higher in those with afistula.

**FIGURE 2:**
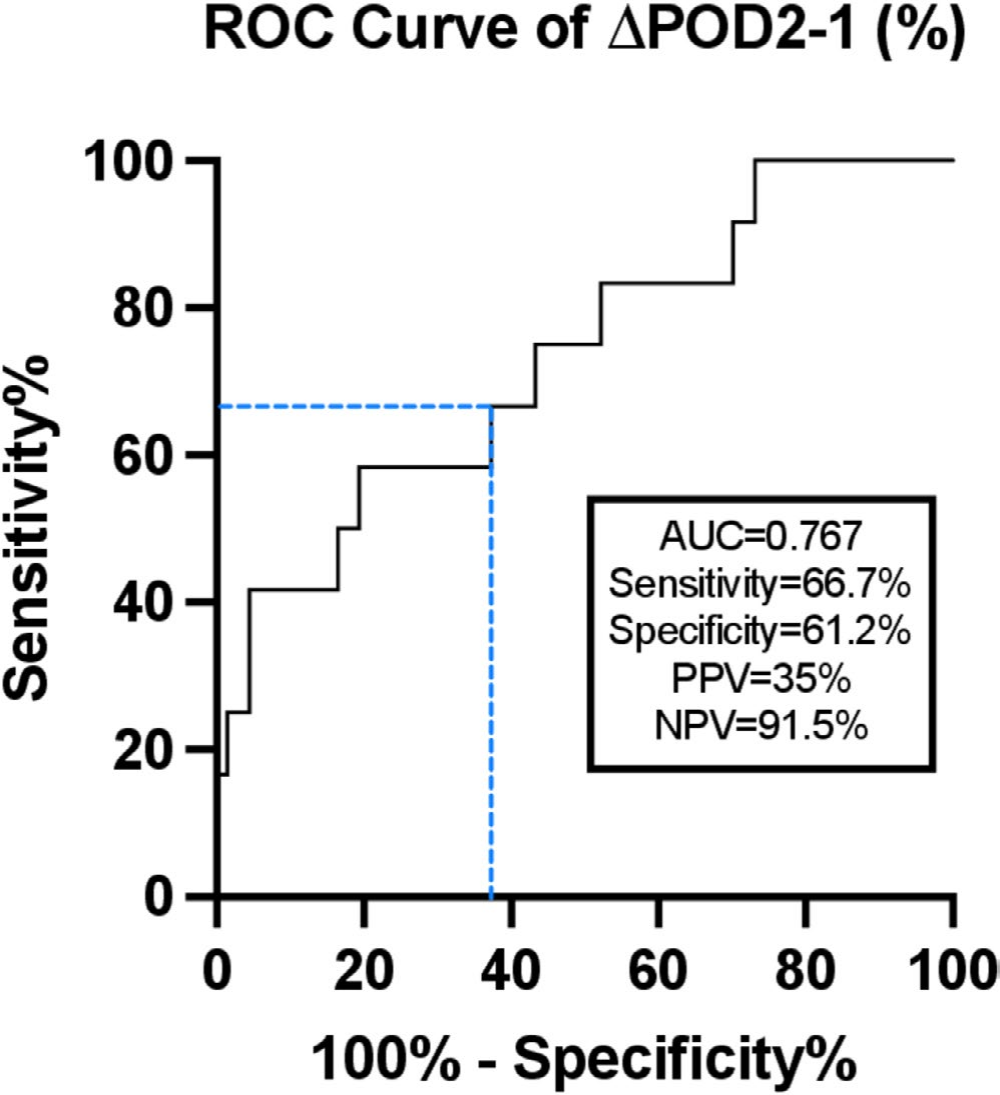
Receiver‐operator characteristic curve for ∆POD2‐1 (%) to predict salivary fistula with an ideal threshold of 15%.

On ROC analysis, ∆POD2‐1 predicted salivary fistula with an optimal threshold of 15% with an AUC of 0.767, sensitivity 58.3% (95% CI = 27.7%–84.8%), and specificity 80.6% (95% CI = 67.4% to −88.1%) (Figure 2). When retrospectively applied to our cohort, a cutoff of 15% resulted in a PPV of 35% (95% CI = 20.4%–49.4%) and NPV of 91.5% (95% CI = 84.3%–95.4%). When ∆POD2‐1 was analyzed as a continuous variable to predict salivary fistula, binary logistic regression revealed an OR of 1.008 (95% CI = 1.003–1.014) to predict salivary fistula (*p* = 0.004). In other words, for every 1% increase in ∆POD2‐1 amylase, a patient would be 1.008 or 0.80% times more likely to have a salivary fistula. Conversely, a 1% decrease in ∆POD2‐1 amylase would correspond to a patient being 0.80% times less likely to develop a fistula. In comparison, ∆POD5‐4 was a poorer predictor with an optimal threshold of 108% (AUC 0.491, sensitivity 27.3%, specificity 96.7%). Given the weak predictive value of ∆POD5‐4, in addition to its worse applicability for identifying salivary fistulas early in the postoperative course, we elected to exclude ∆POD5‐4 from further analysis.

### 3.5 Logistic Regression

**FIGURE 3:**
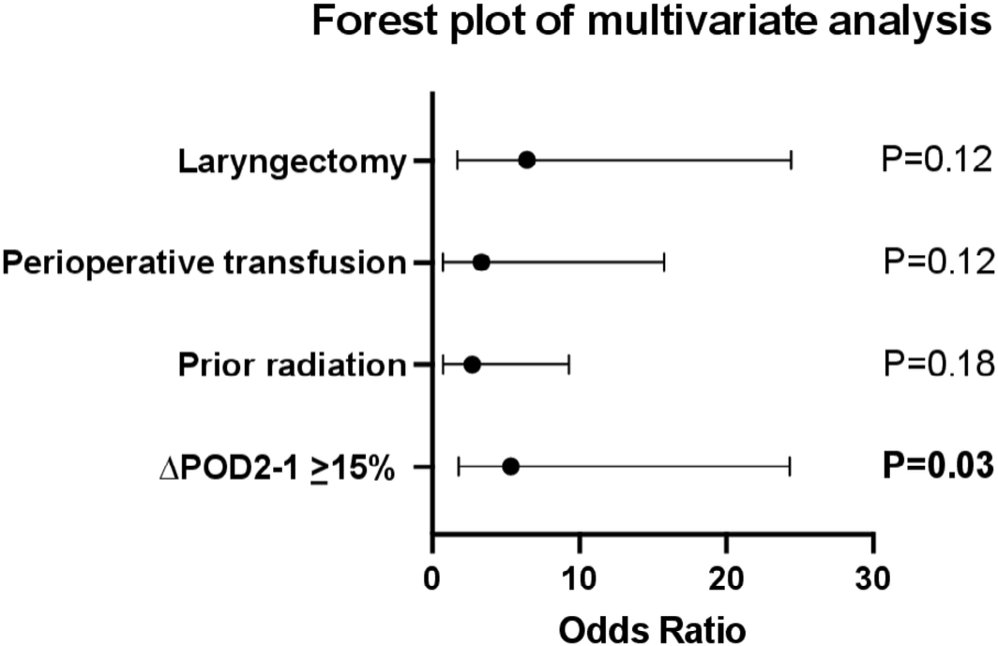
Forest plot of variables on multivariable analysis.

Univariate analyses were conducted on all variables to identify significant predictors of salivary fistula (Table 2). As described in the methods section, variables were included in the multivariate analysis if they had a *p*‐value of < 0.2 on univariate analysis. On univariate logistic regression, ∆POD2‐1 ≥ 15% (OR 5.81, 95% CI 1.59–21.29, *p* = 0.008), prior RT (OR 2.7, 95% CI 0.74–9.28, *p* = 0.13), perioperative transfusion (OR 3.33, 95% CI 0.71–15.75, *p* = 0.13), and laryngectomy (OR 6.44, 95% CI 1.7–24.41, *p* = 0.006) were found to be predictive of salivary fistula. On multivariate analysis, ∆POD2‐1 ≥ 15% remained the only significant predictor of salivary fistula (OR 5.35, 95% CI = 1.79–24.3, *p* = 0.03) when controlling for prior RT (*p* = 0.12), perioperative transfusion (*p* = 0.18), and laryngectomy (*p* = 0.12) (Figure 3).

**TABLE 2:** Univariate logistic regression to predict salivary leak.

Variable	OR	95% CI	*p*‐value
Age	1.03	0.97–1.09	0.35
Gender	0.78	0.19–3.21	0.73
Body mass index (kg/m^2^)	1.01	0.91–1.12	0.9
Preoperative prealbumin	1.01	0.932–1.09	0.85
Tumor category	1.68	0.69–4.11	0.25
Nodal category	1.05	0.62–1.62	0.99
Cardiac comorbidities	2.28	0.46–11.34	0.31
Pulmonary comorbidities	1.16	0.27–4.82	0.84
Vascular comorbidities	0.92	0.1–8.44	0.94
Alcohol abuse	2.19	0.63–7.56	0.22
Smoking history	1.67	0.41–6.78	0.47
Diabetes	2.46	0.55–11.03	0.24
Prior chemotherapy	1.05	0.3–3.67	0.95
Preop hemoglobin	0.88	0.64–1.22	0.46
Preop TSH	0.87	0.3–2.49	0.79
POD1 drain volume	0.49	0.98–1.10	0.49
POD2 drain volume	1.002	0.98–1.03	0.81
POD3 drain volume	1.006	0.99–1.02	0.49
POD4 drain volume	1.03	0.99–1.06	0.31
POD5 drain volume	1.03	0.98–1.08	0.23
Laryngectomy	6.44	1.7–24.41	0.006
Prior radiation	2.7	0.74–9.28	0.13
Perioperative transfusion	3.33	0.71–15.75	0.13
∆POD2‐1 (≥ 15%)	5.81	1.59–21.29	0.008

Abbreviations: ∆POD2‐1, change in drain amylase concentration from POD1 to POD2; CI, confidence interval; OR, odds ratio; POD, postoperative day.

## 4 Discussion

Postoperative salivary fistula remains one of the most significant complications after head and neck free flap reconstruction. In this study, a change in amylase of ≤ 15% from POD1 to POD2 was predictive of *not* developing a salivary fistula during postoperative hospitalization, with a NPV of 91.5%, though sensitivity (58.3%) and PPV (35%) were lacking.

Drain fluid amylase has been utilized at some institutions as an adjunct to clinical examination to indicate fistula formation. However, a lack of data has prevented most head and neck surgeons from modifying clinical management based on amylase alone. To date, there have been 5 studies that have investigated drain amylase, with varying results. Notably, none of these studies investigated changes in amylase. In 1984, Larsen and Schuller examined drain fluid amylase on PODs 1–5 as a predictor of orocutaneous fistula formation in a group of 36 patients (Larsen and Schuller 1984). A downward trend in amylase was observed for those with an uncomplicated postoperative course, whereas an upward trend was seen in those who fistulized (Larsen and Schuller 1984). However, an absolute value could not be correlated with fistula formation, leading the authors to suggest that changes in daily amylase values are more important (Larsen and Schuller 1984). Few studies have attempted to further investigate this marker, leading to confusion regarding its implications. In a group of 87 total laryngectomy patients, Aydogan et al. found drain fluid amylase to be significantly higher on PODs 3–5 in those who formed a pharyngocutaneous fistula, though a predictive threshold value was not identified, limiting the clinical applicability of these findings (Aydoğan et al. 2003) Morton et al. reported on 86 total laryngectomy patients, demonstrating that drain amylase > 4000 IU/L on POD1 was predictive of salivary fistula. Similar to our study, they noted that the predictive ability was better for identifying patients who would *not* have a fistula during postoperative hospitalization (sensitivity 59%, specificity 64%, PPV 29%, NPV 86%) (Morton et al. 2007). Notably, some studies have found drain amylase to be non‐predictive of salivary fistula formation. Kasapoglu et al. did not find any difference in amylase levels on PODs 1–3 in a study of 32 total laryngectomy patients (Kasapoğlu et al. 2009), while Saroul et al. did not identify any difference on PODs 1–2 in a group of 28 total laryngectomy patients (Saroul et al. 2023).

In our study, we found that the change in drain amylase concentration from POD1 to POD2 was a strong *negative* predictor of salivary fistula formation during hospitalization. Similar to the prior literature, this value had a poor sensitivity (58.3%) and PPV (35%) to predict salivary fistula. Rather, the strength of this value was in predicting those who would *not* develop a fistula. A change in amylase of less than 15% between POD1 to POD2 corresponded to a 91.5% NPV and specificity of 80.6%. It can be estimated that for each 10% decrease in amylase from POD1 to POD2, a patient is 1.08 times less likely to develop a fistula. An explanation for the significance of this POD1‐2 change may be that in patients who have watertight intraoperative closure and appropriate wound healing properties, salivary fistula into the neck intraoperatively can be expected to resorb over the next couple of days, whereas those with poor closure may continue to fistula saliva into the neck and soon develop into a clinically appreciable fistula.

The clinical relevance of our variable is that it may be used as an early *negative* predictor of salivary fistula. This may allow some patients to be placed on a “fast track” discharge, which could include early drain removal, trach change, consideration of decannulation, early PO intake, and/or accelerated hospital discharge. Amylase is relatively cheap to measure, with the analysis costing < $10 at our institution. Though a formal cost‐effectiveness analysis was beyond the scope of this study, prolonged LOS is known to increase cost of care, likelihood of complications, work for medical staff, and patient distress. Those with salivary fistulas in the present study had a significantly higher average LOS of 26.4 ± 14.5 days compared to 11.7 ± 4.9 days for those without a salivary fistula. In a study of 264 free flap patients, Lang et al. found that discharge delay occurred in 65% of patients; reasons were most commonly awaiting supplies (38%), case management error (23%), and rejected by facility (23%) (Lang et al. 2019). Moreover, of the patients who experienced discharge delays, 11% experienced complications while awaiting discharge. By modifying discharge criteria in select patients, these delays may be reduced. Notably, we purposefully did not modify clinical management based on amylase so that we could better understand the relationship of amylase with wound outcomes. However, future studies may examine the efficacy of modifying discharge criteria based on amylase levels.

In this study, drain amylase concentrations on PODs 1, 2, 3, 4, and 5 were not statistically different between those who developed a salivary fistula versus those who did not. This may be partially due to the high variability in amylase levels. Levels of amylase production and concentration vary greatly among individuals, and can be attributed to a number of factors including stress, circadian rhythms, oral health, and genetic differences (Carpenter et al. 2017). In their study of laryngectomy patients, Larsen et al. reported that while one patient had a baseline salivary amylase of 460 IU/L, another patient had a baseline salivary amylase of 64,000 IU/L (Larsen and Schuller 1984). This natural variability likely drove the wide ranges of amylase seen in the present cohort. One way to control for these differences is to examine relative changes in drain amylase, rather than absolute values (Larsen and Schuller 1984). Anecdotally, an increase in drain volume is commonly viewed as a possible indicator of a salivary leak. However, in our data, we found that daily JP drain volume as well as changes in drain volume were not statistically different in those with a salivary fistula. This is in line with prior studies which have shown that drain volume does not impact measures of drain amylase concentration (Lu et al. 2020).

Drain amylase in the setting of major pancreatic surgery has been studied extensively. In 2005, the International Study Group on Pancreatic Fistula defined postoperative pancreatic fistula as an amylase level in the drain fluid three times higher than the upper normal serum value on POD3 (Bassi et al. 2005). Since then, the predictive value of amylase concentrations as early as POD1 has been investigated in order to facilitate early drain removal and shorten LOS. In 2007, a group from the University of Verona proposed that patients with drain amylase ≤ 5000 U/L on POD1 carry a risk of postoperative fistula low enough that allows for early drain removal on POD3 (rather than POD5) (Bassi et al. 2010). Since then, other studies have identified various POD1 cutoffs ranging anywhere from 90 to ≥ 4000 IU/L as predictors (Rykina‐Tameeva et al. 2023). Importantly, the emphasis of these studies was on the *negative* predictive value of these cutoffs. Zelga et al. found that POD1 drain amylase carried a low sensitivity and PPV for pancreatic fistula, whereas the NPV of POD1 drain amylase was 98% (Zelga et al. 2015). The authors proposed that the negative predictive value of drain amylase can be used to facilitate early drain removal and accelerate hospital discharge (Zelga et al. 2015). Some authors within the pancreatic surgery literature have suggested that the *change* in drain amylase is more valuable than daily values in isolation. In 2023, Ahmad et al. created a risk analysis calculator to demonstrate that a 70% decrease in amylase from POD 1 to 2 corresponded with a 50% odds reduction of fistula development, regardless of the absolute POD1 value (Ahmad et al. 2023).

The clinical utility and reliability of drain amylase is complicated by many factors. Amylase on POD1 may be artificially high from residual intraoperative salivary contamination, rather than a true fistula. In our cohort, amylase was overall highest on POD1 (2159 ± 5026.4 IU/L). Similar to our findings, drain amylase has been found to be highest on POD1 in prior studies (Kasapoğlu et al. 2009). As previously mentioned, individual differences in baseline salivary amylase concentration and levels may vary widely, complicating the identification of an accurate cut‐off value. Moreover, depending on the location of the surgical drain from which the amylase is measured the drain may not be close to the site of the salivary fistula, resulting in a false negative, or vice versa. Larsen et al. noted that in one of their patients, the drain close to the mucous membrane closure had an initial drain amylase of 4870 IU/L, whereas the second drain was placed further away and had an initial amylase of 232 IU/L (Larsen and Schuller 1984). In our study, we placed a drain along the mucosal closure line and collected all amylase measures from this drain. Additional limitations specific to our study include a smaller sample. Our cohort included a heterogenous cohort of patients with primary tumor sites and subsites of the larynx and oral cavity, though amylase did remain a significant predictor when controlling for laryngeal site on multivariable analysis. A cost‐effectiveness analysis of using drain amylase to allow for early discharge will be important, though this was beyond the scope of our study given that clinical management was not changed based on amylase. Future studies should also follow patients post‐discharge for delayed salivary fistulas.

## 5 Conclusions

Change in surgical drain fluid amylase from POD1 to POD2 was found to be associated with fistula formation following free flap reconstruction of the aerodigestive tract. Importantly, a change in amylase of < 15% from POD1 to POD2 was best at identifying patients who are at low risk of developing salivary fistula during postoperative hospitalization, with a negative predictive value of 91.5%. However, sensitivity (58.3%) and positive predictive value (35%) were lacking, highlighting the continued need for biomarkers that can identify salivary fistulas before they become clinically apparent. Future studies are needed to prospectively examine the efficacy of accelerating clinical discharge based on early postoperative drain fluid amylase.

## Conflicts of Interest

Shaum S. Sridharan is an Editorial Board member of Microsurgery. and a co‐author of this article. To minimize bias, they were excluded from all editorial decision‐making related to the acceptance of this article for publication.

## Data Availability

The data that support the findings of this study are available from the corresponding author upon reasonable request.
